# Real‐world evidence following a mandatory treatment break after a 1‐year prophylactic treatment with calcitonin gene‐related peptide (pathway) monoclonal antibodies

**DOI:** 10.1002/brb3.2662

**Published:** 2022-06-10

**Authors:** Michael Nsaka, Armin Scheffler, Sebastian Wurthmann, Hannah Schenk, Christoph Kleinschnitz, Martin Glas, Dagny Holle

**Affiliations:** ^1^ Department of Neurology and Centre for Translational Neuro‐ and Behavioural Sciences (C‐TNBS), West German Headache Center University Hospital Essen, University Duisburg‐Essen Essen Germany; ^2^ Department of Neurology and Centre for Translational Neuro‐ and Behavioural Sciences (C‐TNBS), Division of Clinical Neurooncology University Hospital Essen, University Duisburg‐Essen Essen Germany

**Keywords:** CGRP (pathway) antibodies, disease modification, guidelines, migraine, one‐year prophylactic treatment, treatment break

## Abstract

**Background:**

Current German and European guidelines suggest migraine patients undertake a treatment break after 9 to 12 months of treatment with CGRP (pathway) monoclonal antibodies.

**Methods:**

Clinical routine data of highly resistant migraine patients were analyzed before treatment with CGRP monoclonal antibodies (baseline), after 12 months of treatment, and following a treatment break between November 2018 and December 2020 in the West German Headache Centre, University Hospital Essen, Germany. Monthly migraine days (MMD), monthly headache days (MHD), and days of acute medication intake (AMD) were assessed.

**Results:**

Complete clinical data from 46 migraine patients (14 episodic migraine (EM), 32 chronic migraine (CM) patients) treated with erenumab (*n* = 40), galcanezumab (*n* = 4), and fremanezumab (*n* = 2) were analyzed. The mean number of MMDs among EM and CM patients after 12 months of CGRP antibody treatment increased during the treatment break by 5.18 (SE 0.92, *p *< .001) and 5.06 (SE 1.22, *p = *.003) days, respectively. There was an increased intake of acute medications among episodic (4.72, SE 0.87, *p *= .004) and chronic migraine patients (3.01, SE 1.08, *p *= .013) during treatment break. Eighty‐three percent of patients (*n* = 38) were dissatisfied with the mandatory treatment break. All patients continued with a CGRP (pathway) monoclonal antibody after the mandatory treatment break.

**Conclusion:**

A mandatory break in CGRP (pathway) monoclonal antibody therapy had a negative short‐term impact on migraine patients.

## Graphical abstract

The manuscript focuses on the relevance of treatment break after twelve months of treatment with the CGRP monoclonal antibodies. Validated data obtained during in depth interviews obtained at the West‐German Headache Center, Essen Germany was analyzed

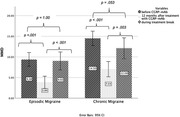

## 1 INTRODUCTION

Calcitonin gene‐related peptide (pathway) monoclonal antibodies (CGRP [pathway] mAbs) are an effective and well‐tolerated treatment option for episodic (EM) and chronic migraine (CM) under real‐world conditions (Bhakta et al., 2021; Diener et al., 2020; Sacco et al., 2019; Scheffler et al., 2020). We analyzed three monoclonal antibodies available on the German market: erenumab, which acts on the CGRP receptor as well as fremanezumab and galcanezumab which target the ligand itself. The European Medicines Agency (EMA) approves all three mAbs for migraine patients with at least four monthly migraine days. In Germany, CGRP (pathway) mAbs are only covered by the statutory health insurance if all first‐line medications (i.e., metoprolol/propranolol, amitriptyline, flunarizine, topiramate, Onabotulinumtoxin A for CM) have shown no effect or could not be used due to side effects or contraindications (Diener et al., 2020).

Preventive migraine treatments aimed at reduction of migraine frequency and severity as well improvement of quality of life are generally recommended in every patient who meet one of the following criteria (Hien & Annika, 2019): patients having four or more headaches a month or at least eight headache days a month, presence of debilitating attacks despite appropriate acute management, difficulty tolerating or having a contraindication to acute therapy, presence of medication‐overuse headache, patient preference or presence of certain migraine subtypes such as hemiplegic migraine, migraine with brainstem aura, migrainous infarction, or frequent, persistent or uncomfortable aura symptoms. For first‐line migraine prophylactics, a treatment break after 6−12 months of therapy is currently recommended to ascertain the necessity of a further prophylactic treatment (International Headache Society, 2004; Silberstein, 2000). Thus, a possible disease modifying effect from the prophylactic medication and/or a “natural” improvement of migraine over the treatment period could be unveiled, as migraine is a cyclic disorder with varying intensity and severity over time (Andreou & Edvinsson, 2019). Furthermore, a treatment break aims at shortening the duration of therapy to prevent associated side effects and reduce treatment costs. Accordingly, current German (Diener et al., 2020) and European (Andreou & Edvinsson, 2019) guidelines suggest a treatment break of CGRP (pathway) mAb treatment after 9 to 12 months to re‐evaluate the effectiveness and necessity of further treatment. In recently available data, patients did not benefit from a treatment break (De Matteis et al., 2021; Gantenbein et al., 2021; Schiano di Cola et al., 2021; Vernieri et al., 2021)^.^

## 2 METHODS

Clinical routine data of 46 migraine patients (14 EM [12 females, 2 males] and 32 CM [27 females, 5 males]) were analyzed at baseline (before initiation of CGRP [pathway] mAb treatment). Patients were treated at the West German Headache Centre, Department of Neurology, University Hospital Essen, Germany between November 2018 and November 2020 and prospectively followed up. The analysis was approved by the independent ethics committee of the University Hospital Essen.

Patients meeting the following criteria were included in the analysis: (a) EM/CM according to the current diagnostic criteria of the International Headache Classification (ICHD‐3), (b) documented 90 days history of monthly migraine days (MMD), monthly headache days (MHD), and monthly intake of acute medication (AMD) preceding initiation of CGRP (pathway) monoclonal antibody using standardized headache dairies of the West German Headache Center, Essen, (c) completion of a 12‐month treatment with a CGRP monoclonal antibody, and (d) presence of a treatment break.

Although a treatment break duration of 3 months was arbitrarily agreed upon, treatment was prematurely reinitiated in patients who suffered severely following cessation of treatment. MMD, MHD, and AMD are reported by all patients (*n* = 46) as average monthly mean values over 90 days before treatment and after 12 months of treatment. MMD (*x*), MHD (*x*), and AMD (*x*) were also determined over the distinct period of treatment break (y) for each patient. Cumulative values obtained from patients during the mandatory treatment break were standardized to monthly equivalents ((*x*/y) × 30). Response rates to changes in concomitant symptoms are as follows: aura (*n* = 34), need for rest (*n* = 46), vertigo (*n* = 46), nausea (*n* = 46), phono‐ and photophobia (*n* = 46), and response to acute medication (*n* = 46). All patients indicated how satisfied they were with a mandatory treatment break (*n* = 46).

Data were analyzed using SPSS software (IBM SPSS Statistics for Windows, Version 27.0. Armonk, NY: IBM Corp). Friedman test of differences among repeated measures was used to compare MMD, MHD, and AMD before, after 12 months of treatment and after the treatment break. Wilcoxon signed‐rank test was conducted as a post hoc test to analyze population mean differences between MMD, MHD, and AMD after 12 months of treatment and during the therapeutic break. The test procedures were two‐sided, and Bonferroni's method for multiple comparison was applied (alpha *=* .05/3). Descriptive statistics was carried out to evaluate changes in concomitant symptoms, acute medication response as well as treatment satisfaction during the treatment break.

## 3 RESULTS

Forty‐six migraine patients (40 patients were treated with erenumab, 4 with galcanezumab, and 2 with fremanezumab) were treated with CGRP (pathway) mAbs. Details regarding demographic data and numbers of MMD, MHD, and AMD before initiation of treatment, after 12 months, and after initiation of the mandatory treatment break are summarized in Table 1.

**TABLE 1:** Patients’ characteristics and cross‐sectional trends during and after CGRP monoclonal therapy

Episodic migraine (*n* = 14)										
Age, y: 49.5 (SD 7.0)female: male: 12:2										Baseline vs. during treatment break
	Before treatment (baseline)	At 12th month, d	50% response (vs. baseline), %	*p*	Change from baseline, d	During treatment break	50% worsening (vs. 12 months), %	Change from 12^th^ month, d	*p*	*p*
MMD (SD)	9.38 (2.88)	3.84 (2.57)	42.9	.001	−5.54 (SE 0.74)	9.02 (3.79)	64.29 (*n* = 9)	5.18 (SE 0.92)	< .001	= 1.00
MHD (SD)	11.69 (2.46)	4.47 (2.30)	35.7	.307	−5.31 (SE 0.87)	11.84(7.36)	64.29 (*n* = 9)	7.37 (SE 1.96)	.013	< .001
AMD (SD)	9.04 (3.02)	3.99 (2.29)	35.7	.001	−5.04 (SE 0.82)	8.71 (3.43)	64.29 (*n* = 9)	4.72 (SE 0.87)	.004	= 1.00

CHRONIC MIGRAINE (*n* = 32)										
Age, y: 48.8 (SD 12.9)female: male: 27:5										Baseline vs. during treatment break
	Before treatment (baseline)	At 12th month, d	50% response (vs. baseline), %	*p*	Change from baseline, d	During treatment break	50% worsening (vs 12 months), %	Change from 12^th^ month, d	p	p
MMD (SD)	14.50 (4.84)	7.03 (5.15)	50.0	< .001	−7.47 (SE 0.88)	12.09 (7.04)	56.25 (*n* = 18)	5.06 (SE 1.22)	.003	.053
MHD (SD)	18.38 (5.49)	12.66 (7.97)	37.5	< .001	−5.72 (SE 1.41)	17.29 (8.21)	46.88 (*n* = 32)	4.62 (SE 1.37)	.004	.401
AMD (SD)	12.78 (5.51)	6.94 (5.42)	40.6	< .001	−5.77 (SE 0.86)	9.73 (6.16)	40.63 (*n* = 13)	3.01 (SE 1.08)	.013	.067

Abbreviations: AMD: days of acute medication intake; MHD, monthly headache days; MMD: monthly migraine days; SD, standard deviation.

Two (4.35%), 15.22% (*n* = 7), 41.30% (*n* = 19) and 39.13% (*n* = 18) of migraine patients treated with CGRP (pathway) mAbs for 12 months showed a 100%, 75% (75–99%), 50% (50–74%), and an insufficient (< 50%) reduction in MMD after 12 months of treatment, respectively. Hereafter, mandatory treatment break lasted 63.60, 47.14, 65.89, and 73.89 days among the above listed efficacy subgroups 100%, 75%, 50%, and insufficient, respectively. MMD (∆ 6.25, SE 1.21, *p* < .001), MHD (∆ 7.54, SE 1.62, *p* < .001), and AMD (∆ 4.26, SE 1.36, *p* = .005) increased significantly during the treatment break only among migraine patients who had previously obtained a 50% (50–74%) reduction in MMD following 12 months of CGRP (pathway) mAb treatment. Changes in MMD, MHD, and AMD among efficacy subgroups are summarized in Table 2.

**TABLE 2:** Significance levels with respect to changes in MMD, MHD, and AMD efficacy subgroups, respectively, during treatment break from calcitonin gene‐related (pathway) monoclonal antibodies

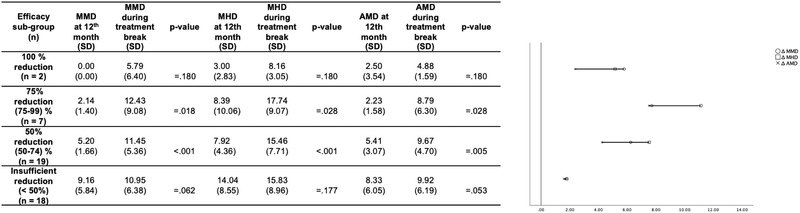

Abbreviations: AMD: days of acute medication intake; MHD, monthly headache days; MMD: monthly migraine days; SD, standard deviation.

To enhance clinical relatability of this study, patients were stratified into EM and CM subtypes. Following 12 months of CGRP (pathway) mAb treatment, there was a significant decrease in MMD among episodic (9.38 vs. 3.84 MMD, *p* = .001) and chronic migraine patients (14.50 vs. 7.03 MMD, *p *< .001). Changes in MHD among episodic (11.69 vs. 4.47 MHD, *p* = .307) migraine patients were insignificant. MHD among chronic migraine patients (18.38 vs. 12.66 MHD, *p* < .001) and AMD (EM: 9.04 vs. 3.99 AMD, *p *= .001; CM: 12.78 vs. 6.94 AMD, *p *< .001) were significantly decreased.

**FIGURE 1:**
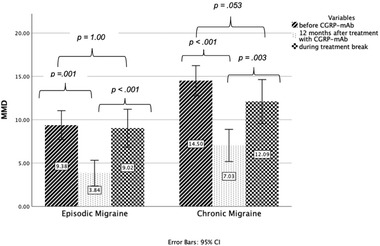
Changes in MMD during and after calcitonin gene‐related peptide (CGRP) monoclonal antibody treatment. *Abbreviation*: MMD: monthly migraine days

The average duration of treatment break in EM and CM patients were 68.88 and 58.92 days, respectively. Sixty‐four percent of EM patients (*n* = 9) reported at least a 50% increase in MMD, MHD as well as AMD during the mandatory treatment break. Fifty‐six percent (*n* = 18), 47% (*n* = 32), and 41% (*n* = 13) of CM patients reported at least a 50% increase in MMD, MHD, and AMD during the mandatory treatment break, respectively. The mean number of MMD was increased by 5.18 (SE 0.92, *p *< .001) among EM patients and by 5.06 (SE 1.22, *p* = .003) among CM patients during the treatment break. The mean number of MHD was increased by 7.37 (SE 1.96, *p* = .013) among EM patients and by 4.62 (SE 1.37, *p *=.004) among CM patients during the treatment break. The mean number of AMD was increased by 4.72 (SE 0.87, *p *= .004) among EM patients and by 3.01 (SE 1.08, *p* = .013) among CM patients during the treatment break (Figure 1).

Pretreatment MMD (EM: *p* = 1.00, CM: *p* = .053), MHD (EM: *p* < .001, CM: *p* = .401), and AMD (EM: *p = *1.00, CM: *p = .067*) did not significantly differ from levels during the mandatory treatment break.

**FIGURE 2:**
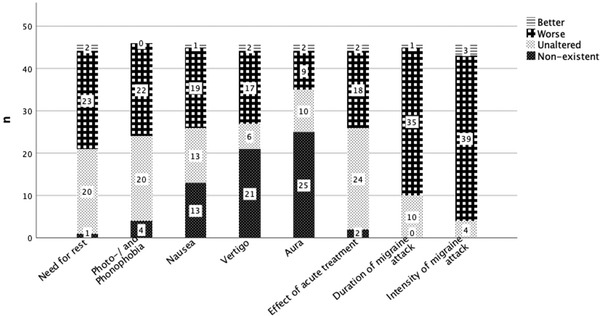
Changes in concomitant vegetative symptoms and efficacy of acute medications during treatment break from calcitonin gene‐related peptide (CGRP) monoclonal antibodies

More than half of patients reported an increase in length (*n* = 35, 76%) and intensity of migraine attacks (*n* = 39, 85%) during the treatment break. Concomitant vegetative symptoms deteriorated less (Figure 2).

Self‐reported general satisfaction with the mandatory treatment break was low (very unsatisfactory/unsatisfactory: 83 %, *n *= 38). All patients continued CGRP‐migraine therapy after the mandatory treatment break.

## 4 DISCUSSION

Our real‐world data suggest that the guideline suggestion of considering a treatment break after a year of treatment with CGRP‐monoclonal antibodies results in a significant worsening of MMD, MHD, AMD, intensity of attacks, length of attacks as well as concomitant symptoms in most migraine patients when compared to levels at 12 months of CGRP (pathway) mAb treatment. Similarly, although our work was not specifically designed to formally assess disease modification, a relevant disease modification did not appear to exist after a year of CGRP‐monoclonal antibody treatment.

The emergence of the CGRP‐monoclonal antibodies, which are the first of a class of migraine prophylactics designed to target specific known mechanisms involved in a migraine attack (the trigeminal pain system), presents a case for a detailed observation of a possible modification of migraine during treatment.

Our data confirm recently published cohort studies by De Matteis et al. (2021), Schiano di Cola et al. (2021), Gantenbein et al. (2021), and Vernieri et al. (2021). De Matteis et al. showed a significant early disease worsening in more than 50% of patients after erenumab discontinuation after a 52‐week treatment in patients with a continuous positive response to the drug (*n* = 32). In a multicentric observational study conducted by di Cola et al., patients who underwent a treatment break from erenumab (17.06 ± 6.5 vs. 4.8 ± 2.5; *p* < .001) reported a significant increase (255%) in MMD. A significant reduction in MMD was observed 3 months after treatment re‐initiation, comparable to the effectiveness before the treatment break. Gantenbein et al. showed, similarly in a cohort study, increased MMD in the first (52.9%, *n* = 34) and second (88.2%, n = 34) months after the CGRP (pathway) mAb treatment break. Surprisingly, the cohort study by Gantenbein et al. showed that in the second month during the mandatory treatment break, patients still had less MMD than before the initial start of mAb treatment (EM: 9 ± 5 [*n* = 19] vs. 14 ± 7 [*n* = 31]) and CM: (8 ± 4 [*n* = 15] vs. 20 ± 5 [*n* = 21]). This suggests a possible residual effect after discontinuation of the CGRP (pathway) mAb treatment or a disease modification. Raffaelli et al. (2019), in a similar fashion, encourages discussions on the concept of a possible disease modification or a residual effect after discontinuation of the CGRP (pathway) mAb treatment. In this work, Raffaelli et al. observed no significant difference in monthly migraine days during a 12‐week observation period after open‐label termination when compared to the last 4 weeks of preventive erenumab and galcanezumab treatment of chronic migraine patients (*p* = .228, *n* = 16). Our results, though methodologically different from the above listed studies, showed no significant modification in MMD, MHD, or AMD after a year of treatment.

Like our findings, a significant disease modification during a 5‐month follow‐up interval after a 6‐month double‐blind treatment period could not be established in the EVOLVE‐1 and −2 studies (Stauffer et al., 2019). In these studies, there was a significant increase in monthly migraine days in the first post‐treatment month in patients previously treated with 120 mg (*p *< .01) and 240 mg (*p *< .001) of galcanezumab. The quality of life among patients previously treated with galcanezumab also worsened significantly.

Similarly, Andreou et al. (2018) observed that 91% of episodic migraine patients reported a significant worsening in migraine, which necessitated a re‐initiation of treatment after voluntarily discontinuation of treatment with Onabotulinumtoxin A. Noteworthily, the investigated migraine patients in this study showed previously no satisfactory response to first‐line prophylactic medications before initiation of treatment with Onabotulinumtoxin A. Also, 34% of the 200 patients (*n* = 68) who underwent the voluntary treatment discontinuation from Onabotulinumtoxin A had been reclassified from a chronic to episodic migraine during the treatment, which indicates a good treatment response before the treatment break.

An important contrasting study to ours was the double‐blinded PROMPT study (Diener et al., 2007). In this study, Diener et al. demonstrated a possible disease‐modifying effect during a treatment break following a 26‐week open‐label phase treatment with topiramate. A striking finding observed during the treatment break was that even though an increase in MMD was reported, a significant sustained benefit existed among migraine patients after 6 months of treatment with topiramate, as MMD did not return to pretreatment baseline values (*p <* .0001). This benefit was attributed to a possible repair of an existing central neuronal dysfunction implicated in migraine‐pathophysiology. These results matched those observed in earlier studies with migraine preventive treatments such as flunarizine (Nuti et al., 1996; Wöber et al., 1991) and beta‐blockers (Wöber et al., 1991).

Our study also investigated changes in MMD, MHD, and AMD among various efficacy subgroups. Although patients who obtained a 50–64% reduction in MMD during CGRP (pathway) mAb‐treatment reported a significant increase in MMD, MHD, and AMD during the mandatory treatment break, surprisingly, patient subgroups who benefitted even more from CGRP (pathway) mAb‐treatment (≥ 75% response rate) reported no significant change in MMD, MHD, and AMD. This inconsistency may be due to the relatively small sample size among efficacy subgroups who obtained more than a 75% treatment benefit. We also observed that patients who obtained a less than 50% reduction in MMD during treatment with CGRP (pathway) mAbs did not show a relevant increase in MMD, MHD, and AMD during the mandatory treatment break. The lack of significant change among this subgroup of patients initially showing a less than 50% reduction in MMD during treatment with CGRP (pathway) mAbs could be either attributed to a chronic resistance to treatment or the presence of another headache subtype that responds less favorably to CGRP (pathway) mAbs treatment.

It remains uncertain to what extent long‐term treatment with CGRP (pathway) mAbs results in an over‐ or under‐expression of CGRP receptors. Clinical effects of these are equally yet to be seen. Future studies should focus on ascertaining an optimal timing when patients best benefit from a treatment break. Furthermore, we are presently short of sufficient data regarding the course of migraine after re‐initiation of CGRP (pathway) mAb after a treatment break. This should also be an interest of future investigations as a relative improvement or worsening of symptoms compared to CGRP‐mAb‐naïve states are possibilities hereafter.

The findings in our report are subject to six limitations. First, a possible nocebo effect after an unvoluntary discontinuation of effective treatment cannot be ruled out. Further data are needed from patients who terminate treatment on their own volition or in the context of a placebo‐controlled study. Secondly, it remains unclear to what extent patients who have high frequencies of migraine attacks, yet not affected by a chronic migraine or another headache subtype, will fare after discontinuation of CGRP (pathway) mAbs. Another weakness of our study was its monocentric nature. In addition, an assessment of the quality of life during the treatment break is needed. Furthermore, a standardization of MMD, MHD, and AMD according to the duration of the treatment break (as a measure of average disease burden spread over 3 months) may not be an adequate estimate for evaluation of real‐world changes in patients' frequency, as some patients may still have a greater than 50% response for example in the first month and a worsening hereafter. This, however, was necessitated as upholding of safe ethical standards was a priority. Compelling patients to involuntary undergo a 3‐month treatment break would have been otherwise unethical and unsafe. A fifth limitation of this study was that the number of subjects who obtained a 100% and 75% reduction in MMD during the treatment were relatively less than in other groups (50%, < 50%). While one might argue that a 100% and 75% reduction in MMD in 9 out of 46 highly resistant migraine patients does not necessarily come as a major surprise, these might be poorly considered single groups and is another possible limitation. Finally, our data were largely obtained from patients treated with erenumab. More data are required especially regarding CGRP ligand antibodies (galcanezumab and fremanezumab).

## 5 CONCLUSION

A mandatory generalized break of CGRP (pathway) mAb treatment after 1‐year results in a worsening of an already improved migraine when compared to CGRP (pathway) mAb‐naive states. This procedure currently suggested in the guidelines caused a high level of dissatisfaction among most patients and thus should be critically re‐assessed.

## AUTHOR CONTRIBUTIONS

Michael Nsaka has the major role in the acquisition of data, analyzed, interpreted the data, and drafted the manuscript for intellectual content. Armin Scheffler analyzed and interpreted the data and revised the manuscript for intellectual content. Sebastian Wurthmann, Hannah Schenk, Christoph Kleinschnitz, and Martin Glas interpreted the data and revised the manuscript for intellectual content. Dagny Holle designed and conceptualized study interpreted the data and drafted the manuscript for intellectual content. All authors read and approved the final manuscript.

## CONFLICT OF INTEREST

Michael Nsaka received travel fees from LICHER MT. AS received travel fees from Teva, honoraria for participation on an advisory board of Novartis. SW reports personal fees from Allergan, personal fees from Teva, personal fees from Novartis, outside the submitted work. HS declares no conflict of interest. Christoph Kleinschnitz has received honoraria, a consulting or advisory role to declare for Alexion, Almirall, Amgen, Amicus, Bayer, Biogen, Biotronik, Boehringer Ingelheim, Biontech, Bristol Myers‐Squibb, Celgene, CSL Behring, Daiichi Sankyo, Desitin, Eisai, Ever Pharma, GE Healthcare, Johnson & Johnson, MedDay Pharmaceuticals, Merck Serono, Mylan, Novartis, Pfizer, Roche, Sanofi‐Genzyme, Siemens, Stada, Stago, and Teva. Martin Glas received honoraria from Novartis, UCB, Teva, Bayer, Novocure, Medac, Merck, Kyowa Kirin, has a consulting or advisory role to declare for Roche, Novartis, AbbVie, Novocure, and Daiichi Synkyo, and received travel fees from Novocure and Medac. Dagny Holle has received scientific support and/or honoraria from Biogen, Novartis, Lilly, Sanofi‐Aventis, Teva, Allergan, Hormosan.

### PEER REVIEW

The peer review history for this article is available at https://publons.com/publon/10.1002/brb3.2662.

## Data Availability

The datasets used and/or analyzed during the current study are available from the corresponding author on reasonable request.
